# Wnt activation as a potential therapeutic approach to treat partial limbal stem cell deficiency

**DOI:** 10.1038/s41598-023-42794-8

**Published:** 2023-09-21

**Authors:** Clémence Bonnet, Sheyla González, Sophie X. Deng, Jie J. Zheng

**Affiliations:** 1 Department of Ophthalmology, Stein Eye Institute, David Geffen School of Medicine, University of California, Los Angeles, 100 Stein Plaza, Los Angeles, CA 90095 USA; 2 INSERM, UMRS1138, Team 17, From Physiopathology of Ocular Diseases to Clinical Development, Cordeliers Research Center, Ophthalmology Department, Cochin Hospital, AP-HP, Université Paris Cité, 75005 Paris, France; 3 Molecular Biology Institute, University of California, Los Angeles, CA 90095 USA

**Keywords:** Cell biology, Stem cells, Medical research

## Abstract

Limbal epithelial stem/progenitor cells (LSCs) are adult stem cells located at the limbus, tightly regulated by their niche involving numerous signaling pathways, such as Wnt. Wnt proteins are secreted morphogens that play critical roles in embryonic development, stem cell proliferation, self-renewal, tissue regeneration, and remodeling in adults. It has been shown that a small molecule Wnt mimic could improve LSCs expansion ex vivo. Damage to the LSCs and/or their niche can lead to limbal stem cell deficiency (LSCD), a condition that can cause corneal blindness and is difficult to treat. This study explored if repopulating residual LSCs in partial LSCD through Wnt activation could be a novel therapeutic approach. To mimic LSCD due to a chemical injury, single cultured LSCs were exposed to various concentrations of sodium hydroxide. A progressive loss of the LSCs phenotype was observed: the percentage of p63^bright^ cells and cytokeratin (K)14^+^ cells decreased while the percentage of K12^+^ increased. Wnt activation was attained by treating the LSCs with lithium chloride (LiCl) and a small-molecule Wnt mimic, respectively. After 18 h of treatment, LSCs proliferation was increased, and the LSCs phenotype was recovered, while the untreated cells did not proliferate and lost their phenotype. The percentage of p63^bright^ cells was significantly higher in the Wnt mimic-treated cells compared with untreated cells, while the percentage of K12^+^ cells was significantly lower. These findings suggest that local Wnt activation may rescue LSCs upon alkaline injury.

## Introduction

The limbus, i.e., the junction between the cornea and the conjunctiva, contains a population of self-renewing adult stem cells called limbal stem/progenitor cells (LSCs)^1^. The LSCs are responsible for the maintenance of the integrity of the corneal surface and for the continuous renewal of the corneal epithelium^2^. The LSCs closely interact with their microenvironment, referred to as the LSC niche, consisting of stromal cells, melanocytes, and extracellular matrix, in a highly vascularized and innervated stroma^3,4^. Damage or traumatism to the LSCs and/or their niche can lead to limbal stem cell deficiency (LSCD), a condition that can cause corneal blindness^5,6^. In the severe stage of the disease, when the visual axis is affected and the residual LSCs function cannot be promoted medically, autologous LSC transplantation is one of the cell therapies that can be offered to restore the ocular surface^7^. However, long-term success outcomes of autologous LSC transplantation are only about 75%^8–10^. Allogeneic LSC transplantation can also be offered in bilateral severe to total LSCD. Yet, long term outcomes are lower than autologous LSC transplantation, and allogeneic transplantation requires lifelong oral immunosuppression, limiting its indications^10^. Other cell therapies are being developed, such as the use of oral mucosal epithelial cells or mesenchymal stem cells, given their anti-inflammatory and immunomodulatory properties on damaged tissues^11^.

The diagnosis of the disease now relies on precise in vivo evaluation of the LSC function using anterior segment imaging, such as anterior-segment optical coherence tomography and in vivo laser scanning confocal microscopy^5^. Basal cell density decreases in eyes with LSCD both in the central cornea and the limbus, showing a negative correlation with the severity of the disease; eyes with moderate and severe LSCD can still exhibit some LSCs^12–14^. Despite their altered cell morphology^14^, these remaining cells might still be functional. Indeed, ocular surface optimization of ocular comorbidities, including treatment of ocular surface inflammation, blepharitis, and dry eye, can be sufficient to improve the function of LSCs^7^. These topical therapies can include low doses of topical steroids, autologous serum, punctal plugs, and preservative-free artificial tears^7^. Improvement of the LSCs function in some of these eyes suggests that those residual LSCs in partial LSCD can be repopulated. However, currently, there is no topical therapy available to promote the recovery of partial LSCD by specifically facilitating the repopulation of residual LSCs to increase the success of medical treatment. Such therapy could also prevent the deterioration of partial LSCD to total LSCD. Therefore, there is an unmet need for the development of new medical therapies for partial LSCD that could further reduce the indications of LSC transplantation. Direct and cultivated autologous LSCs transplantations have been shown to reach approximately 70% long-term outcomes^9^. The transplant can currently be performed only for unilateral disease and can be repeated only a limited number of times to ensure the safety of the unaffected eye. Therapies not requiring surgery and that could be repeated as deemed necessary would have the major advantage of alleviating these limitations. To this end, Wnt signaling activators are targets of interest, as Wnt signaling pathway plays a major role in LSC regulation^15–22^. A previous gene profiling study revealed that the pathway is upregulated in the limbus when compared to the central corneal levels^18^. MFH-ND, a bioengineered Wnt activator, has been previously shown to activate Wnt/β-catenin signaling pathway and promote LSCs proliferation in vitro^23^. Moreover, a recent study also showed that LSCs can secrete their own Wnt signaling molecules in an autocrine manner^24^. Finally, Wnt activators can work in synergy with endogenous Wnt signaling to trigger the signalosome assembly^25,26^. By promoting optimal function and proliferation of residual LSCs present in partial LSCD, Wnt signaling activators could be a novel approach to medically managing LSCD.

In this study, we developed an in vitro LSCs alkali burn model, as chemical burns remain one of the most frequent causes of LSCD worldwide^27^. After validation of the model, we explored the effects of Wnt signaling activation on the damaged LSCs and showed that the use of a Wnt mimic could restore the LSCs phenotype and promote their proliferation. The Wnt mimic is a promising new therapeutic option for partial LSCD.

## Results

### Residual LSCs can be found in partial LSCD secondary to chemical burn

**Figure 1:**
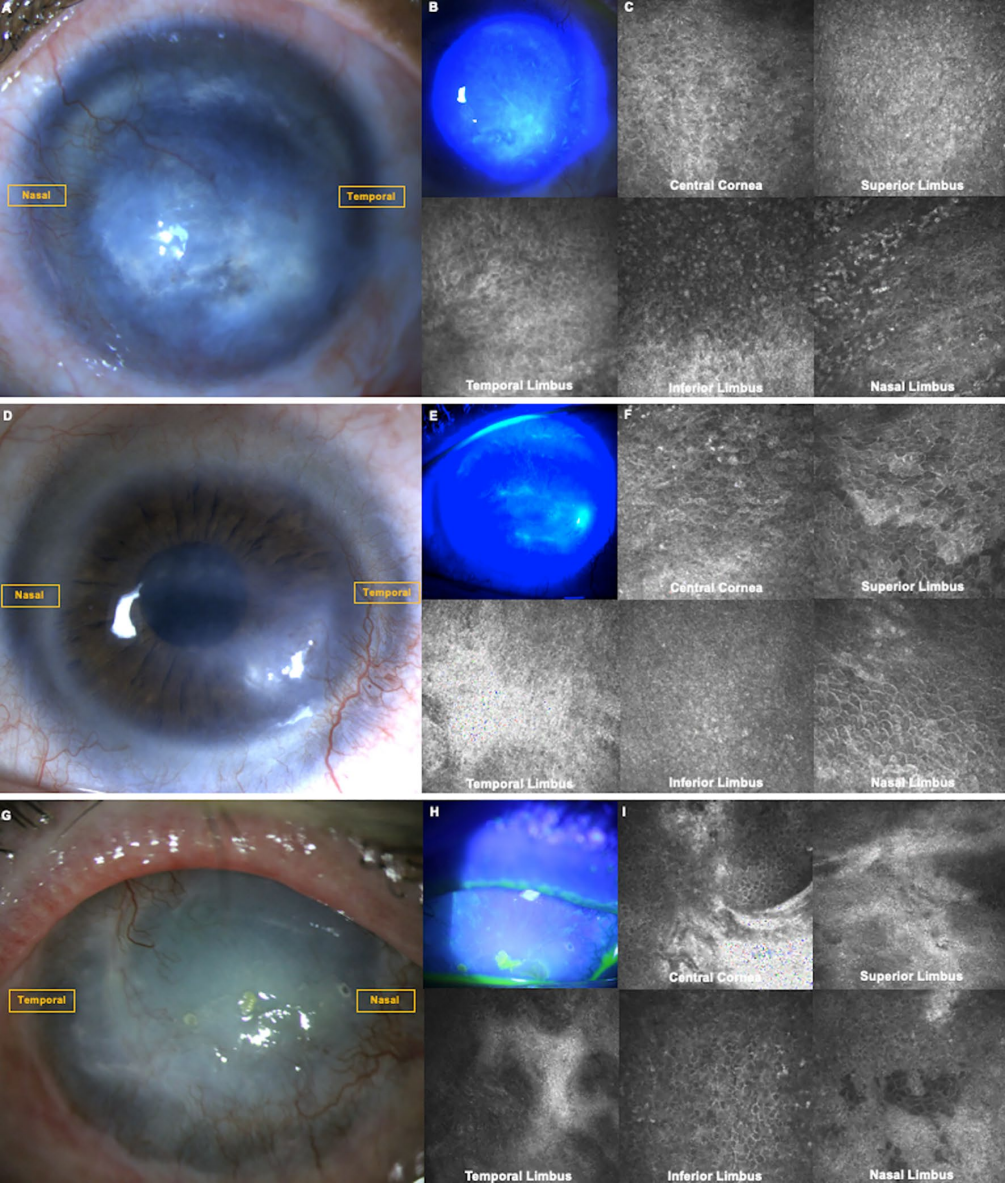
Slit lamp picture with fluorescein staining and in vivo laser scanning confocal microscopy of three eyes with severe partial LSCD. (**A**) Slit lamp picture of an eye with severe LSCD from chemical injury. (**B**) Fluorescein staining and blue cobalt light showing a diffuse vortex staining involving all the cornea corresponding to a partial severe LSCD, stage IIB^5^, score 8 points^28^. (**C**) IVCM confirming the diagnosis of severe LSCD. The central BCD is decreased but residual corneal and limbal epithelial cells can be seen in the central cornea and limbal quadrants, respectively. (**D**) Slit lamp picture of an eye with severe partial LSCD from prolonged contact lens wear. (**E**) Fluorescein staining and blue cobalt light showing a diffuse vortex staining involving all the cornea corresponding to a partial severe LSCD, stage IIB^5^, score 8 points^28^. (**F**) IVCM confirming the diagnosis of severe LSCD. The central BCD is decreased but residual corneal and limbal epithelial cells can be seen in the central cornea and limbal quadrants, respectively. (**G**) Slit lamp picture of an eye with severe partial LSCD from chemical injury 36 years prior. (**H**) Fluorescein staining and blue cobalt light showing a diffuse vortex staining corresponding to a partial severe LSCD, stage III^5^, score 10 points^28^. (**I**) IVCM confirming the diagnosis of severe LSCD. The central BCD is decreased but residual corneal and limbal epithelial cells can be seen in the central cornea and limbal quadrants, respectively, suggesting that this eye had partial severe LSCD.

Figure 1 shows the clinical presentation (Fig. 1) of three eyes with severe unilateral LSCD presenting with residual LSCs by in vivo laser scanning confocal microscopy (IVCM). The first case (Fig. 1A–C) is an eye with LSCD from a chemical burn that occurred twelve years prior. The patient presented with severe LSCD in 2009, confirmed by IVCM. The IVCM images show morphologic epithelial changes compatible with metaplasia^14^. The patient had been treated with ocular surface optimization for three months without improvement. Subsequently, an allogeneic LSC transplantation was performed. The transplantation quickly failed, and the patient was lost in follow-up. The patient returned twelve years later; the patient had a clinical presentation compatible with partial severe LSCD (Fig. 1A,B, corresponding to a stage IIB)^5^, score 8^28^. IVCM was performed to stage the disease and showed residual limbal epithelial cells with decreased central corneal basal cell density (BCD, 5383 cells/mm^2^, Fig. 1C), suggesting that the eye still contained residual LSCs likely from the host, given that the allogeneic transplantation had failed twelve years prior. No nerves were detected, and the basal central corneal and limbal cell morphologies were altered with metaplastic changes (grade 3, Fig. 1C). Additionally, normal limbal structures harboring LSCs, such as palisades of Vogt^29^ or limbal lacunae^30^, were lacking in the affected areas (Fig. 1C). The ocular surface was optimized using preservative-free topical steroids four times a day, artificial tears as needed, resorbable punctal plugs, and epilation of trichiasis. After three months, no improvement was noted clinically. IVCM was repeated, showing no inflammatory cells on the ocular surface following ocular surface optimization but no improvement in biomarkers evaluation^31^. The second case (Fig. 1D–F) is a 49-year-old woman referred to our center for corneal scarring requiring a penetrating keratoplasty (Fig. 1D). She had been treated by simple limbal epithelial transplant (SLET) five years prior for LSCD due to prolonged contact lens wear. The clinical examination shows sub-epithelial scarring, superior, temporal, and nasal corneal neovascularization. The fluorescein staining shows a diffuse vortex staining with epithelial irregularity in temporal, corresponding to a stage 2B (score 8) of LSCD (Fig. 1E). The IVCM scans shows residual metaplastic central corneal epithelial cells (Fig. 1F) with a BCD of 5491 cells/mm^2^, and residual limbal epithelial cells in superior and nasal, while no cells were detected in inferior and temporal. These findings suggest that this eye is suffering from partial LSCD in which the remaining epithelial cells are not sufficient to maintain a healthy ocular surface. The third case (Figs. 1G–I) is a 57 year-old man who suffered a chemical injury in the right eye 36 years prior. At his first visit, he presented with total corneal opacification and neovascularization (Fig. 1G) and diffuse vortex staining (Fig. 1H) compatible with a stage 3, score 10 LSCD. The IVCM scans (Fig. 1I) showed metaplastic corneal epithelial cells in the central cornea with a BCD of 2297 cells/mm^2^. Residual limbal epithelial cells with normal morphology were seen in all four limbal quadrants suggesting that this eye in fact had partial severe LSCD. These three cases show that eyes presenting with partial severe LSCD can exhibit residual limbal epithelial cells. Despite intense ocular surface optimization, allogeneic LSCs transplant in the first case and SLET in the second case, promoting residual LSC function was unsuccessful. These three patients were offered an autologous ex-vivo LSC transplantation, as part of an ongoing clinical trial looking at the safety and efficacy of ex-vivo LSCs transplantation in the management of unilateral LSCD (NCT03957954)^7^. Alternative emerging techniques include the use of non-epithelial cell transplants (oral mucosal graft, embryonic stem cells, induced pluripotent stem cells, MSCs). Growth factors revitalizing the limbal niche are also explored, such as the use of plasma and platelet-rich growth factors^32,33^.

### In vitro model of partial LSCD by chemical burn

To explore the effects of a chemical burn on LSCs, we developed an in vitro model of partial LSCD. Limbal epithelial cells were cultured on a 3T3 cell feeder layer, and their phenotype was confirmed as previously described^20,34^. Briefly, the LSCs phenotype is defined as high expression of p63 (p63^bright^ cells), expression of the stem cell markers cytokeratin 14 (K14), and small size (≤ 12 μm). Differentiation is marked by the expression of the mature corneal epithelial cell marker cytokeratin 12 (K12). Lack of culture contamination by limbal stromal cells is confirmed by the presence of less than 15% of Vimentin-positive cells^20^. More than 80% of pancytokeratin-positive cells confirm the epithelial phenotype of LECs. After 10–12 days in 3T3 co-culture, colonies of LSCs were harvested, seeded on 96 well plates, and exposed to sodium hydroxide (NaOH).

**Figure 2:**
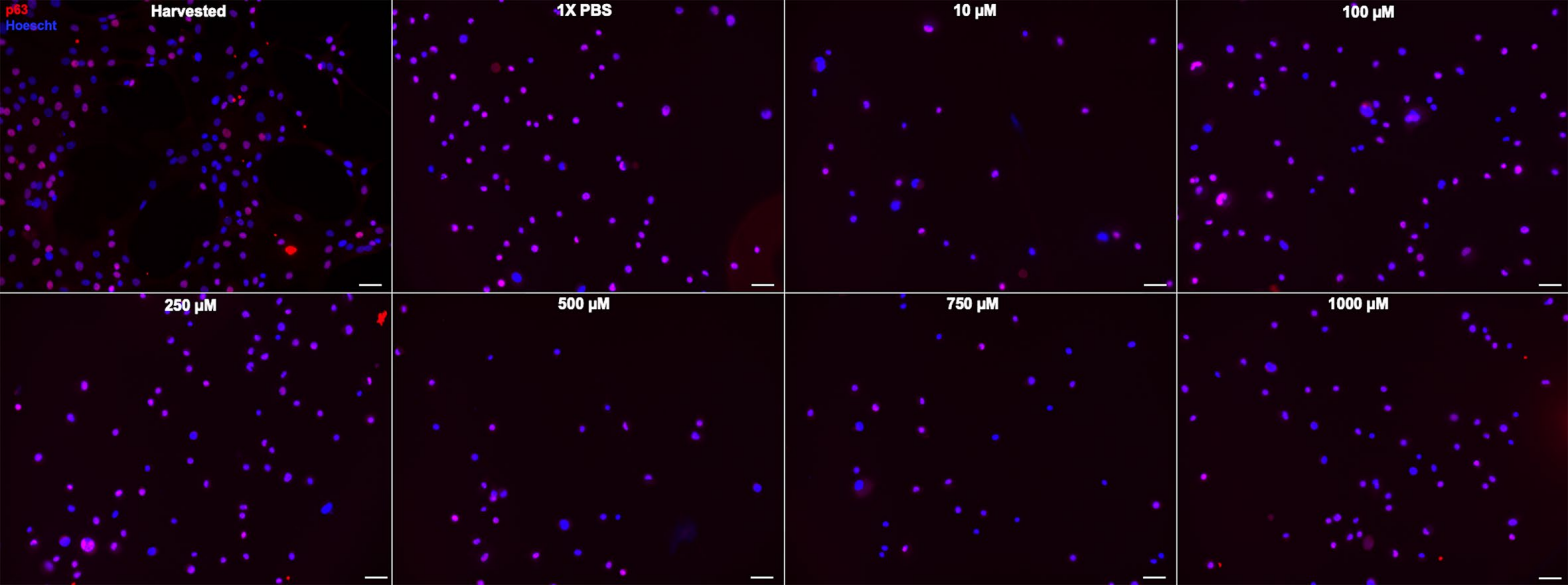
In vitro model of limbal stem cell deficiency: p63 immunostaining. Cultured LSCs exposed for one minute to increasing concentrations of sodium hydroxide, progressively lost their p63^bright^ cells phenotype. Scale bar 20 µm.

**Figure 3:**
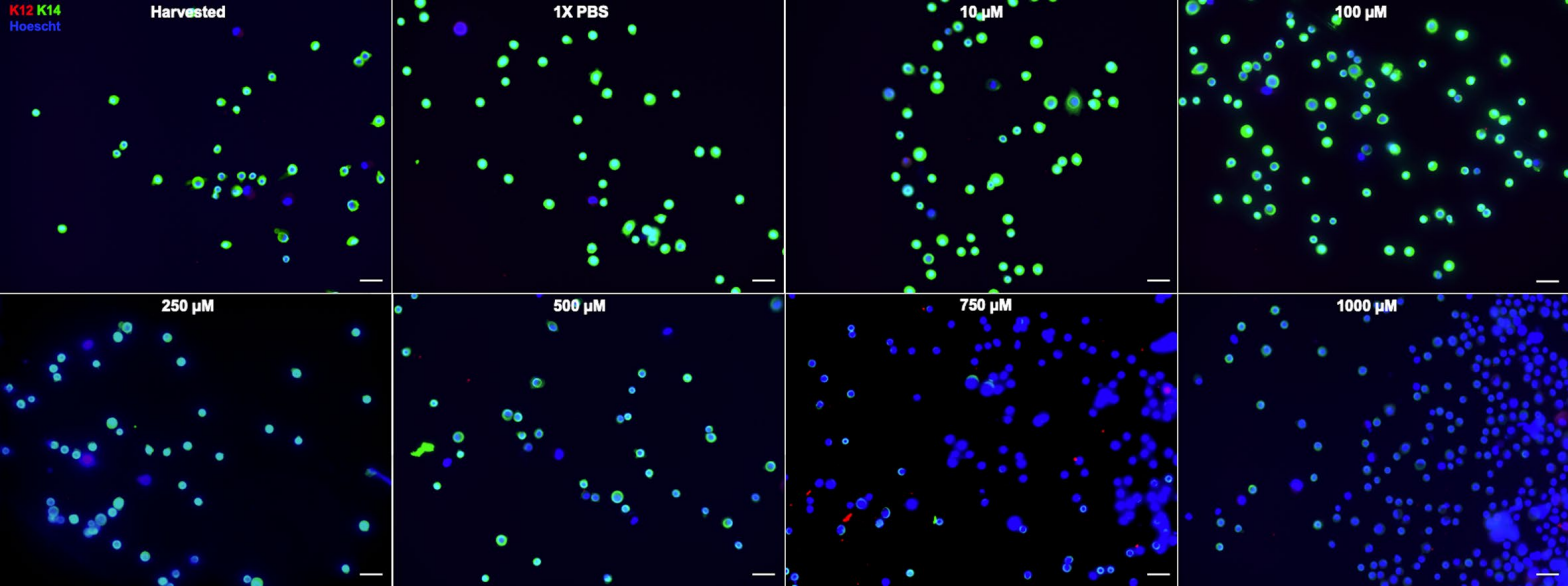
In vitro model of limbal stem cell deficiency: K12/K14 immunostainings. Cultured LSCs exposed for one minute to increasing concentrations of sodium hydroxide, progressively lost their K14 + phenotype. The percentage of K12 + positive slightly increased but the difference was not significant. Scale bar 20 µm.

**Figure 4:**
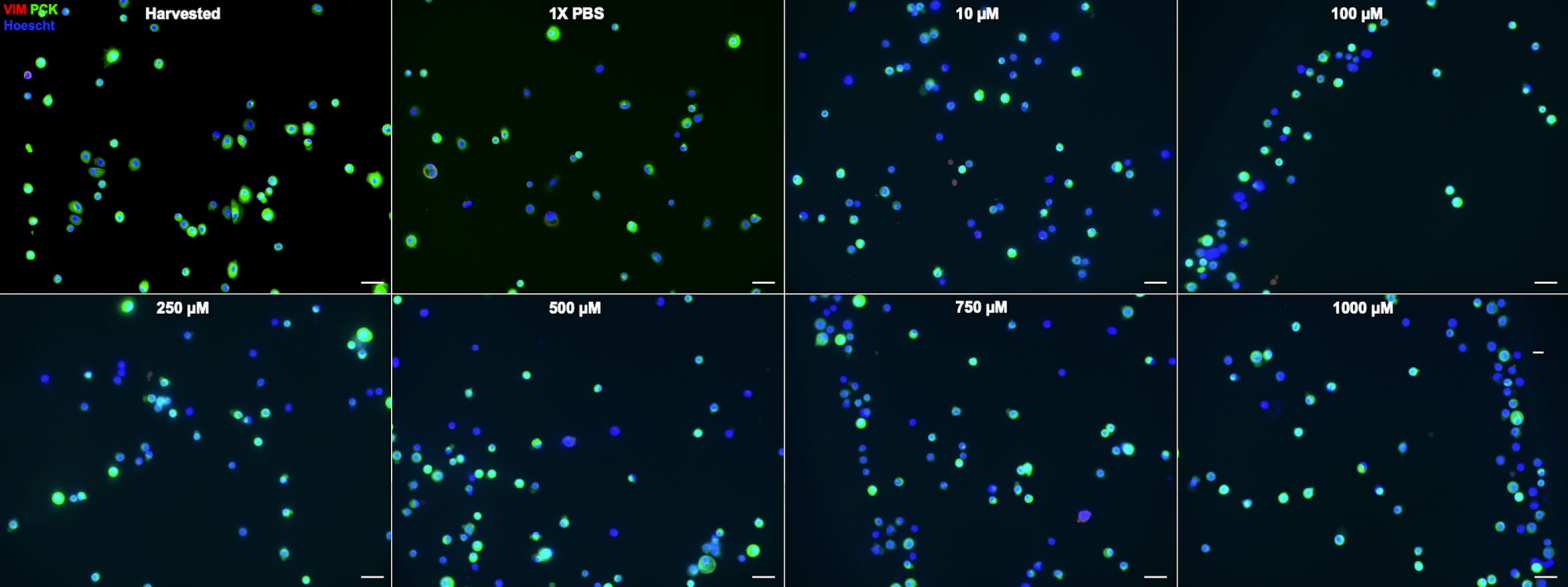
In vitro model of limbal stem cell deficiency: PCK/Vimentin immunostaining. Cultured LSCs exposed for one minute to increasing concentrations of sodium hydroxide, progressively lost their PCK positive phenotype. Scale bar 20 µm.

**Figure 5:**
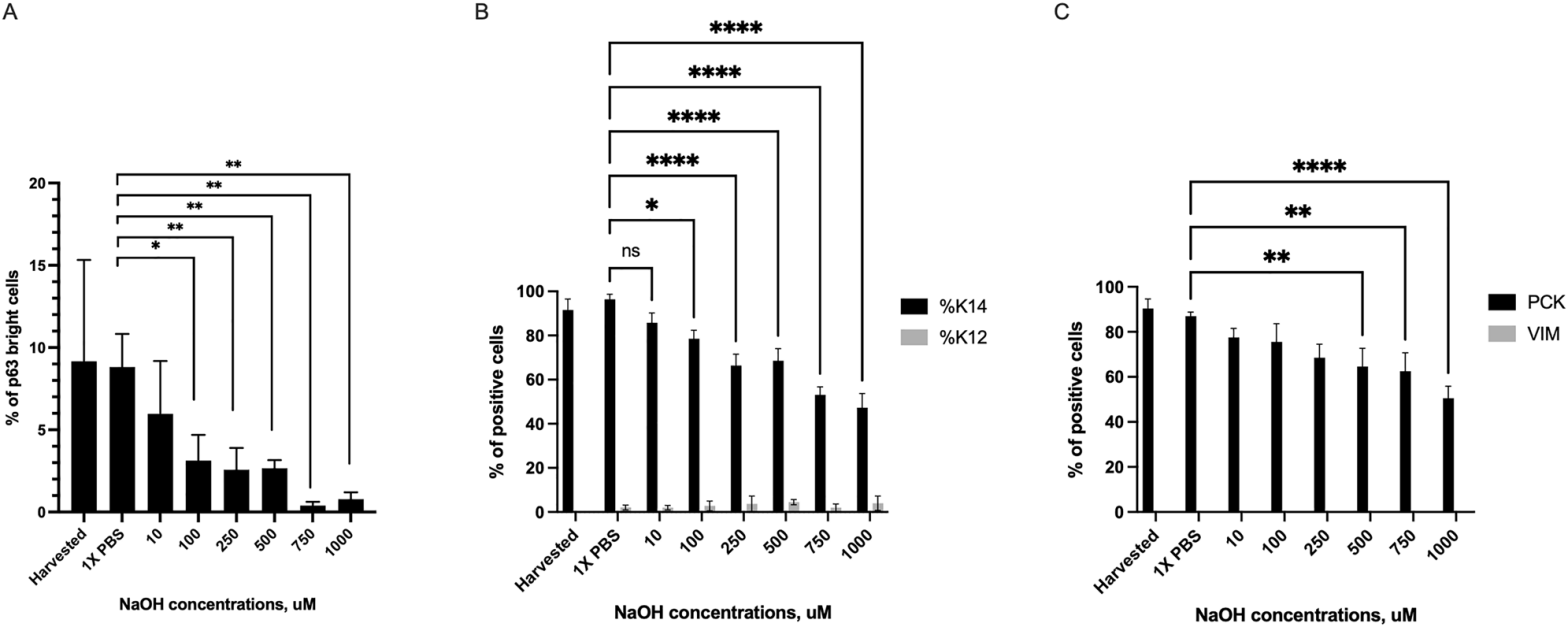
In vitro model of limbal stem cell deficiency: immunostaining quantification. (**A**) The decrease in the percentage of p63^bright^ cells was statistically lower starting at 100 µM NaOH (*p* < 0.05). (**B**) The decrease in the percentage of K14 + cells was statistically lower starting at 100 µM NaOH (*p* < 0.05). The percentage of K12 + positive slightly increased but the difference was not significant. (**C**) The decrease in the percentage of PCK positive cells was significant starting at 500 µM of NaOH (*p* < 0.05).

Residual LSCs are present in partial LSCD, and they are likely functional^12–14,28,35^. To mimic in vivo partial LSCD, we hypothesized that increasing concentrations of NaOH would lead to progressive harm to the LSCs to complete cell death. Therefore, we used increasing concentrations of NaOH solution to determine the threshold concentration necessary to partially harm LSCs (Supplemental Fig. 1). This dose escalated exposure resulted in a progressive loss in LSCs phenotype (Figs. 2, 3, 4, 5). The reduction in the percentage of p63^bright^ cells (Fig. 2) and of K14 + cells (Fig. 3) was significant, starting at 100 µM of NaOH up to 1 M (*p* < 0.05, Fig. 5). The percentage of K12 + cells (Fig. 3) slightly increased with increasing concentrations of NaOH but was not significant (Fig. 2C). The reduction in the percentage of pancytokeratin (PCK) + cells (Fig. 4) was significant for 500 µM, 750 µM, and 1 M of NaOH (*p* < 0.05, Fig. 5), while no vimentin (VIM) + cells were detected. Based on these results, 500 µM was chosen to test the efficacy of the small molecule in all future experiments.

### Treatment with a Wnt mimic can restore the LSCs phenotype after in vitro chemical injury and promote LSCs proliferation

**Figure 6:**
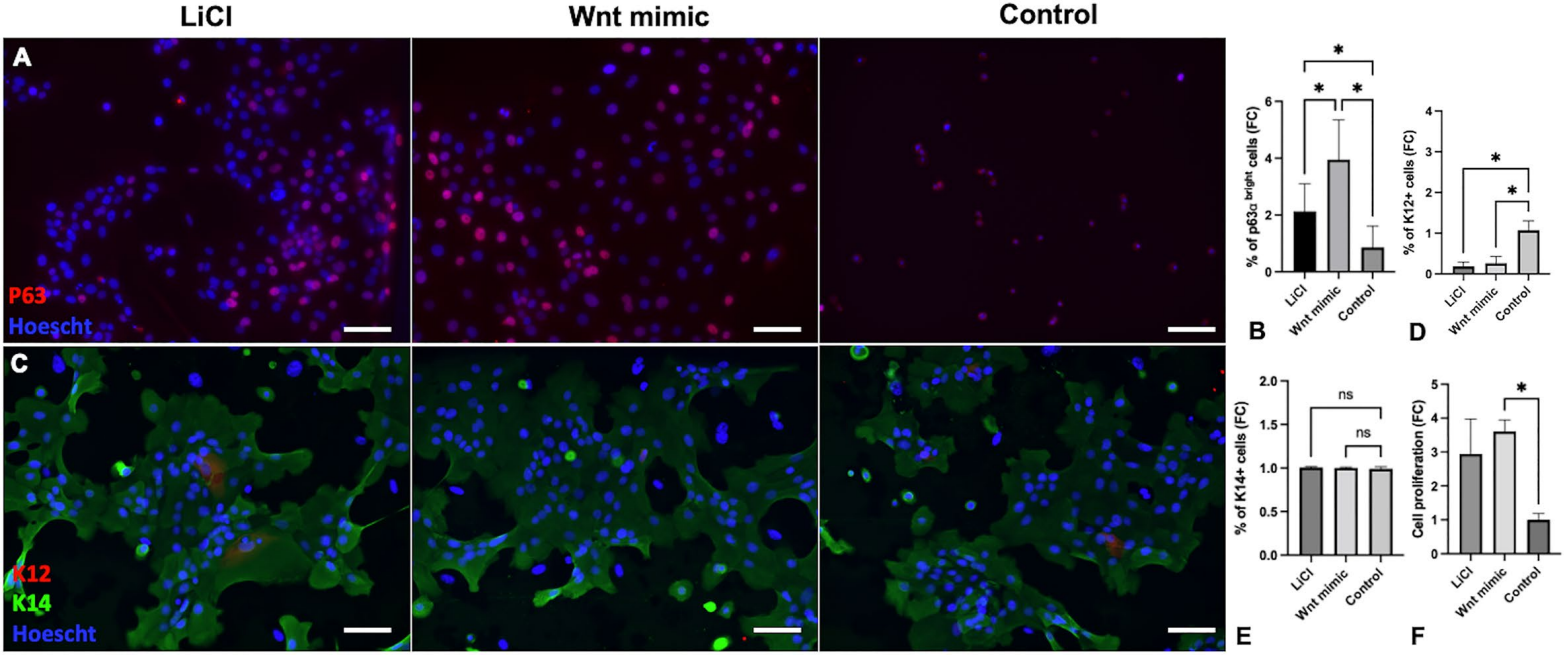
Limbal epithelial cell phenotype after treatment by Wnt activators. (**A**) Immunostainings of p63^bright^ cells after treatment by Wnt mimic, LiCl, or SHEM after chemical burn. (**B**) Treatment with Wnt mimic significantly increased the percentage of p63^bright^ cells compared with the control and LiCl (*p* < 0.05). (**C**) Immunostaining of K12 and K14 cells after treatment by Wnt mimic, LiCl, or SHEM after chemical burn. (**D**) The percentage of K12 + cells was significantly lower in the Wnt mimic and LiCl group (*p* < 0.05) (**E**) The percentage of K14 + cells was comparable in all groups of treatment. (**F**) Cell proliferation was significantly increased in the Wnt mimic group compared to the control (*p* < 0.05). Data are expressed as fold change of expression compared with the unburned SHEM control. One way ANOVA with multiple comparisons and Dunnett post-hoc test. Mean ± SEM of 4 individual experiments. Scale bar 20 µm.

Cultured LSCs were exposed to 500 µM NaOH for one minute and subsequently treated with a Wnt mimic 50 µM, lithium chloride (LiCl) 500 µM, or control-supplemented hormonal epithelium medium (SHEM) for eighteen hours. The concentrations of the Wnt mimic^23^ and LiCl^20^ used were determined based on our previous publications; in the dose-dependent experiments, those concentrations had the best effect in promoting ex-vivo LSC expansion^20,23^. The LSCs phenotype was restored both in the Wnt mimic and in the LiCl group (Fig. 6A). The percentage of p63^bright^ cells was significantly higher compared with the control (*p* < 0.05) and LiCl (4.12 folds increase in the Wnt mimic, 2.17 in LiCl, *p* ≤ 0.05, Fig. 6B).

Comparison between Wnt mimic, LiCl, and the control group showed comparable percentages of K14 + cells (Fig. 6C,E). The maintenance of LSCs is further demonstrated by the significant decrease detected in K12 + cells in the Wnt mimic and LiCl treatment groups (Fig. 6C,D). Cell proliferation was also significantly increased in the Wnt mimic group compared with the control (*p* < 0.05, Fig. 6F). Altogether, these results demonstrate that the Wnt mimic supports the recovery and proliferation of LSCs phenotype after chemical injury in an undifferentiated state.

## Discussion

The role of LSCs in the maintenance of corneal epithelium homeostasis and wound healing has been confirmed in several experimental models showing that a certain number of functional LSCs is necessary to sustain corneal epithelium physiology^36,37^. Below this threshold, losses of both the limbal barrier and corneal epithelium renewal occur, leading to LSCD. Management of LSCD remains difficult because no treatment can directly replenish dysfunctional or deficient LSCs. Moreover, patients with LSCD very often have ocular surface comorbidities that compromise or exacerbate the dysfunction of residual LSCs and threaten the survival of transplanted LSCs^7^. Therefore, optimization of the ocular surface and treating all associated comorbidities can enhance the survival of residual LSCs. It is the first step in the treatment of any case of LSCD^7^. In partial LSCD, where residual LSCs are present, LSC function can sometimes be medically restored without the need for surgical treatment^38^. On the opposite, severe to total LSCD usually requires further ocular rehabilitation using LSC transplantation, as the threshold necessary to restore corneal homeostasis and limbal barrier cannot be reached by medical therapies only^7^. To objectively determine the severity of the disease, it is recommended to perform IVCM and measure BCD^12^, sub-basal nerve density (SND)^35^, and cell morphology^14^. A decrease in BCD and SND is correlated with the severity of the disease^31^. Increasing morphologic alterations, including metaplastic cells to complete loss of epithelial phenotype, are correlated with the severity of the disease. The LSC niche is also very often altered in severe to total cases^39^.

Chemical burn commonly leads to severe or even total LSCD^27^. However, as shown in the case study (Fig. 1), twelve years after the chemical burn injury, despite permanent damage to the LSC niche, the eye can still be classified by the IVCM biomarkers as partial LSCD, suggesting that the eye still contains residual viable LSCs, as described in the literature^12,31^. Therefore, instead of an autologous LSC transplantation, we reason that it may be possible to treat the disease by re-populating those residual LSCs.

In vitro assays will be the first step in exploring such medical therapies, and an in vitro model of partial LSCD is needed. A few chemical/ultraviolet burn in vitro models using immortalized corneal epithelial cell lines have been reported to study corneal wound healing^40–43^, but none of them explored the effects of a toxic injury on primary LSCs. However, given the pivotal role of LSCs in corneal epithelial renewal after injury, following the widely accepted X, Y, Z hypothesis by Thoft and Friend^44^, it is critical to understand the molecular mechanisms of LSCs’ damage following chemical injury. To address this issue, we developed an in vitro model of partial LSCD, focusing on the molecular effects of the burn on the cells, removing the 3T3 feeder layer (i.e., the in vitro LSCs niche). The progressive loss of LSCs phenotype upon exposure to increasing NaOH concentrations confirms that LSCs are susceptible to alkaline injury, as seen in corneal epithelial cell lines^40–43^. We hypothesized that this loss of phenotype could correlate with the morphologic findings seen by IVCM. In such a scenario, in vivo LSCs, losing their stem cell properties following the injury would partially lose their function, failing to maintain the corneal epithelial renewal and limbal barrier, leading to LSCD. We chose 500 µM, which is in the range of a previous report for corneal epithelial cells^42^, corresponding to a loss of approximately 70% of p63^bright^ cells, 30% of K14 positive cells, 8% of PCK positive cells, the first significant change observed, and the peak of increased cell death (Supplemental Fig. 1) as a threshold for partial LSCD, so that the model would not permanently damage the LSCs.

GSK-3β inhibitors are commonly used to activate Wnt/β -catenin signaling pathway^18^, and we show that LiCl, a widely used GSK-3β inhibitor, indeed increases the proliferation rate and the percentage of p63^bright^ cells, suggesting that Wnt activation is a promising approach to re-populate residual LSCs. Nevertheless, although β-catenin is the major player in canonical Wnt signaling pathway, activated Wnt signaling also affects other cellular components^21^. Consistent with this notion, here, we show that, compared to LiCl, used as a control for Wnt activation, a small molecule Wnt mimic more effectively increases the proliferation rate of LSCs and the percentage of p63^bright^ cells in the in vitro partial LSCD model^45^.

LSCs express multiple Wnt ligands^15^, and it is also known that different Wnt ligands can cooperate and synergize for stem cell proliferation^25,26^. Moreover, LSC proliferation and Wnt activation are dose and time-dependent upon exposure to exogenous Wnt in vitro^22^, and a Wnt gradient exists within the limbal epithelium^24^. Therefore, adding exogenous Wnt in case of a partially altered limbal microenvironment could potentially have a synergetic effect with the residual endogenous Wnt ligands remaining in vivo. This may explain why the small-molecule Wnt mimic works better than the GSK-3β inhibitor. In either case, our study shows that it may be possible to promote the proliferation and repopulation of the residual LSCs in partial LSCD through Wnt activation. Therefore, those small-molecule Wnt activators may have the potential to be developed as novel therapeutic reagents to treat partial LSCD and ultimately alleviate the need for LSCs transplantation. It has also been shown that canonical Wnt signaling activation plays a role in corneal wound healing^46^, confirming the critical role of Wnt signaling pathway in corneal physiology. In conclusion, this pilot study is the first step towards the use of Wnt small molecules to treat LSCD. To further explore the effect of Wnt activation on LSC stemness and proliferation in the presence of the niche microenvironment, future directions could include the development of a three-dimensional ex-vivo corneal LSCD chemical burn model.

## Methods

### In vivo laser scanning confocal microscopy

In vivo laser scanning confocal microscopy is used to confirm the diagnosis of LSCD at the Stein Eye Institute^12–14,28,35^. Scans are performed in the central cornea and the four limbal areas (superior, inferior, nasal, and temporal) to confirm the diagnosis of LSCD^13^. A minimum of three high-quality Z-scans are acquired in each area using the HRT III with Rostock Cornea module (Heidelberg Engineering GmBH, Germany). The protocol was approved by the Institutional Review Board at the University of California, Los Angeles (UCLA, IRB #10-001601). Appropriate informed consent was obtained from study subjects per IRB protocol. The study was compliant with the HIPAA regulations and adhered to the Declaration of Helsinki.

Several biomarkers are used to confirm the diagnosis and stage its severity^31^. A decrease in the central corneal and limbal BCD, the corneal SND, and the central corneal and limbal epithelial thickness (CET) correlate with the severity of the disease^31^. The BCD is obtained following the manufacturer instruction for cell density calculation, as previously described^12,13^. Briefly, a frame containing at least 50 cells is chosen. All the cells visible within the frame are manually counted by two independent observers. The average corresponds to the BCD. The central corneal and limbal basal cell morphology are also altered: cells become metaplastic in the moderate stage, and a loss of epithelial phenotype occurs in the severe stage. These morphologic changes correlate with the severity of the disease^14^.

### Human corneoscleral tissue

Four human corneoscleral tissues from healthy donors (age 34 to 60 years) were obtained from CorneaGen (Seattle, WA). All tissues were preserved in Optisol-GS (Chiron Ophthalmics, Inc., Irvine, CA) with a death-to-preservation time of less than eleven hours. Experiments using human tissues adhered to the tenets of the Declaration of Helsinki. The experimental protocol was exempted by the University of California, Los Angeles Institutional Review Board (IRB#12-000363).

### Isolation and culture of human LSCs

Limbal epithelial cells (LECs), which included LSCs, were isolated as previously described^47^. Briefly, corneoscleral rims were digested with 2.4 U/mL Dispase II (Roche, Indianapolis, IN) in supplemented hormonal epithelium medium (SHEM) for two hours at 37 °C. The limbal epithelial cell sheets were isolated and digested with 0.25% trypsin and 1 mM EDTA (Life Technologies) for 5 min at 37 °C to obtain a single-cell suspension. Single LECs were seeded at a density of 200 cells/cm^2^ on subconfluent NIH-3T3 mouse fibroblasts. Growth of the NIH-3T3 cells was arrested by treatment with 4 µg/mL of mitomycin C (Sigma-Aldrich; St Louis, MO) for two hours at 37 °C.

SHEM consisted of DMEM/F12 medium (Gibco, Carlsbad, CA, USA) supplemented with 5% fetal bovine serum (FBS, Life Technologies), 1% of N2 supplement (Life Technologies), 2 ng/mL of epidermal growth factor (EGF; Life Technologies), 8.4 ng/mL of cholera toxin (Sigma-Aldrich, St. Louis, MO), 0.5 µg/mL of hydrocortisone (Sigma-Aldrich), 0.5% of dimethyl sulfoxide (DMSO; Sigma-Aldrich), 1% of penicillin/streptomycin (Life Technologies), and 0.2% of gentamicin/amphotericin B (Life Technologies). The medium was changed every 2–3 days, and cells were cultured for ten to twelve days.

### Chemical burn model and treatment

At the end of the culture period, the 3T3 feeder layer was removed by three washes of PBS. The total removal of the PBS was checked under a light microscope. Then, cultured colonies were incubated for one hour in SHEM growth medium supplemented with 2.4 U/mL Dispase II (Roche) at 37 °C. Single cells were obtained after incubation in 0.25% trypsin and 1 mM EDTA (Gibco) for five minutes at 37 °C. To decipher the effect of the burn on the LSCs alone, LSCs harvested were seeded in 96 well plates and placed at 37° for two hours at a density of 30 000 cells/well. Cell attachment was confirmed by the absence of cell movement during gentle agitation of the plate under a light microscope. After washing with 1X PBS three times, the cells were treated with 100 µL of the indicated sodium hydroxide (NaOH) concentration (10 µM to 1 mM in distilled water) or 1X PBS for one minute as previously described^42^.

After model validation, we chose to expose the LSCs to 500 µM NaOH for the subsequent experiments. This concentration is in the range of previous reports for corneal epithelial cells^42^. This concentration corresponds to a loss of approximately 70% of p63^bright^ cells, 30% of K14 positive cells, and 8% of PCK positive cells and was used as a threshold for partial LSCD.

After exposure to 500 µM NaOH for one minute, the cells were washed three times with 1X PBS (200 µL) and incubated with either MFH-ND 50 µM^23^ in SHEM, LiCl at 500 µM^20,45^ or SHEM alone for eighteen hours. The cells were then rinsed three times and immediately fixed for immunofluorescence. Each experiment was performed in triplicates and repeated 4 times with different cornea donors.

### Immunofluorescence stainings

Cells in a 96-well plate were fixed with 4% paraformaldehyde at room temperature for fifteen minutes, washed three times with 1X PBS, blocked, and permeabilized with 1X PBS containing 1% bovine serum albumin (BSA) and 0.5% Triton X-100 (Sigma-Aldrich) for thirty minutes at room temperature. Wells were incubated with primary antibodies in 1X PBS containing 1% BSA and 0.1% Triton X-100 overnight at 4 °C. After incubation, cells were washed with 1X PBS and incubated with the secondary antibodies at 20 °C for one hour. Nuclei were labeled with Hoechst 33342 (Invitrogen, 33342, 4 µg/mL) at 20 °C for fifteen minutes and mounted in Fluoromount medium (Sigma-Aldrich).

Many proteins are preferentially expressed in basal limbal epithelial cells as identified through differential transcriptome studies^48,49^ and reported as putative LSCs markers. One widely used marker is ΔNp63α, an isoform of the transcription factor p63 that has been implicated in stem cell regulation^50^ and is critical for epithelial development and regeneration^51^. Among the six p63 isoforms, ΔNp63α isoform is the most highly associated with LSCs^52^. Detection of ΔNp63α in LSCs is challenging because most of the commercially available antibodies lack specificity and recognize different isoforms. Given that ΔNp63α is the most abundant isoform in the limbus^51–53^, a pattern of the high level of expression detected by the antibody recognizing p63α is considered to mainly represent the pattern of ΔNp63α expression. Moreover, a high proportion of cells (> 3%) expressing a high level of p63α (classified as p63α^bright^ cells) among cultured limbal epithelial cells has been shown to positively correlate with the clinical success of LSC transplantation^9^. Therefore, we used the expression level of p63α^bright^ cells indicated by the intensity of fluorescent antibody staining to estimate the proportion of LSCs in culture^15,20^.

Cytokeratin 14 (K14) is one of the cytokeratins associated with undifferentiated epithelial cells in the human limbal epithelium and is therefore considered a marker of undifferentiated corneal epithelial cells, including LSCs and progenitor cells^54^. K14 expression is mainly found in the basal and suprabasal layers of the limbus in adults^55^. Upon migration to the cornea, the expression of K14 is lost, and K12 is expressed^56^. Additionally, LSCs lack K12, a cytokeratin that marks differentiation seen in more mature cells. Therefore, to distinguish the cytokeratins profile in LSC culture, we used the percentage of K14 + cells as a marker of undifferentiated cells and the percentage of K12 + cells as a marker of mature corneal epithelial cells. Supplemental Table 1 summarizes the primary and secondary antibodies used. Supplemental Fig. 2 provide the immunostainings in the control group.

### Immunofluorescence staining analysis

Images were taken with a Keyence BZ-X710 inverted microscope (Osaka, Japan), and quantitation of expression of markers was performed by using the BZ-X analyzer software (version 1.3.0.3) with the macro hybrid cell-count function. The percentage of positive cells was automatically analyzed using the Keyence BZ-X710 inverted microscope with the BZ-X analyzer software. By specifying the mask area, the software tracked information about multiple parameters, such as the intensity of fluorescence signals in different channels, cell counts, and target area measurements^57^. For p63, the threshold defining a bright cell was manually chosen based on the appearance of typical bright cells in the control group. For K12, K14, PCK, and Vimentin, the threshold was chosen to define a positive cell when membrane fluorescence was detected by the software, regardless of the intensity in the control group. The same thresholds were applied for all conditions tested.

### Cell death and proliferation assay

For model validation, cell death was assessed using propidium iodide (Cat. P3566, ThermoFisher) following the manufacturer’s instruction: one drop of PI and one drop of Hoechst 33342 (Cat. R37605, ThermoFisher) were added and incubated for 30 min. The cells were washed three times with PBS and immediately imaged using the Keyence BZ-X710 inverted microscope. Three individual images were acquired for each donor for each condition. The percentage of positive dead cells was averaged for each condition.

Cell proliferation was assessed as the ratio of cell nucleus counted after eighteen hours of treatment over the number of cells counted after the chemical burn before the treatment from immunostaining pictures acquired in triplicates from different picture areas.

### Statistical analysis

Analysis of variance with multiple comparisons and Dunnett post-hoc test was performed using Prism v. 9.3.1. Bar graphs represent the mean ± SEM of four individual experiments performed in triplicates. A *p*-value ≤ 0.05 was considered statistically significant.

### Supplementary Information

Supplementary Figure 1.

Supplementary Figure 2.

Supplementary Table 1.

## Data Availability

All data and materials are available upon reasonable request.
