# Supplementary figures and images for: Wnt activation as a potential therapeutic approach to treat partial limbal stem cell deficiency
Source: Sci Rep. 2023 Sep 21;13:15670. doi: 10.1038/s41598-023-42794-8 (PMC10514048; doi:10.1038/s41598-023-42794-8)

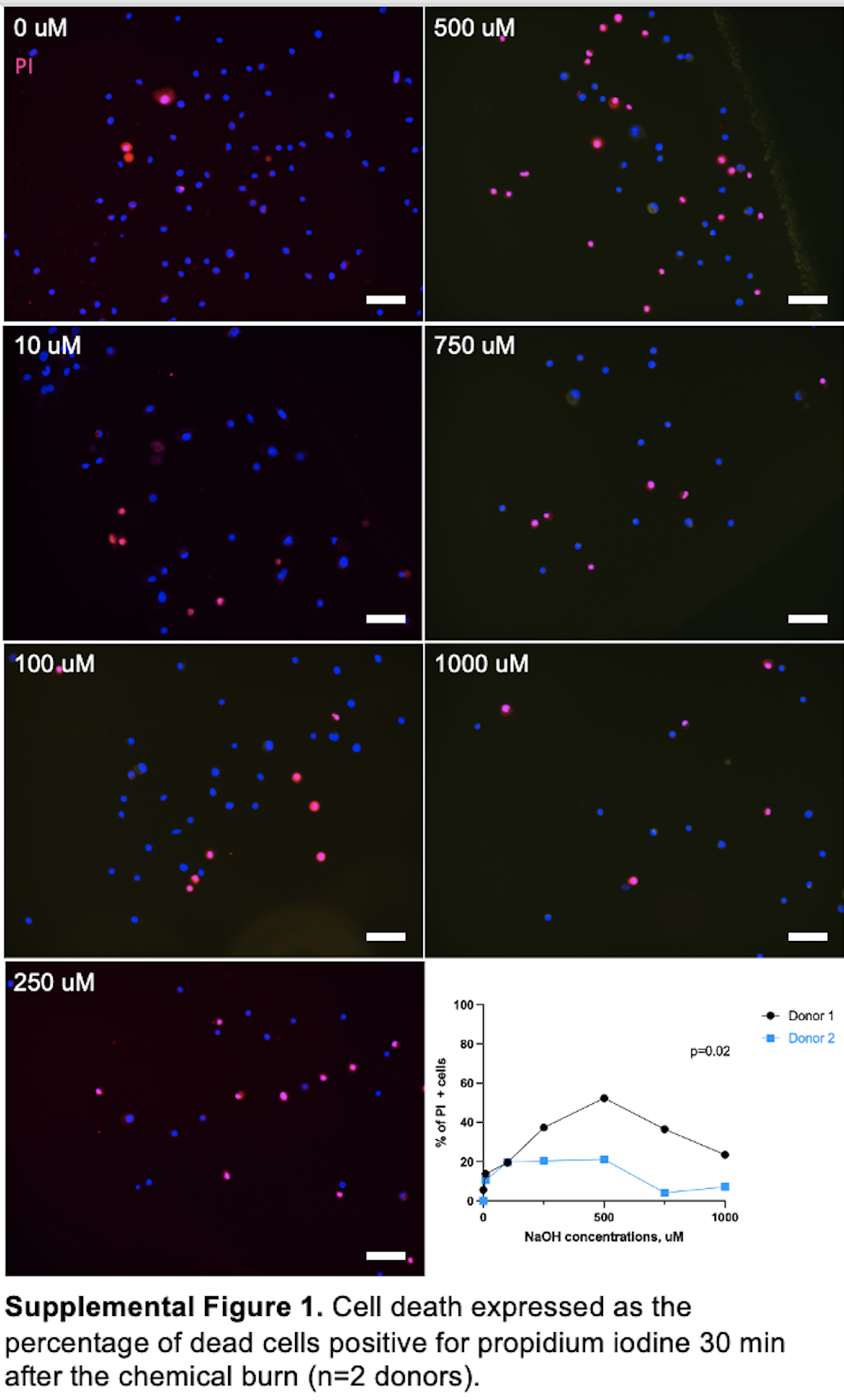

Supplement: Supplementary file 1 — Supplementary Figure 1. [file 41598_2023_42794_MOESM1_ESM.tiff]

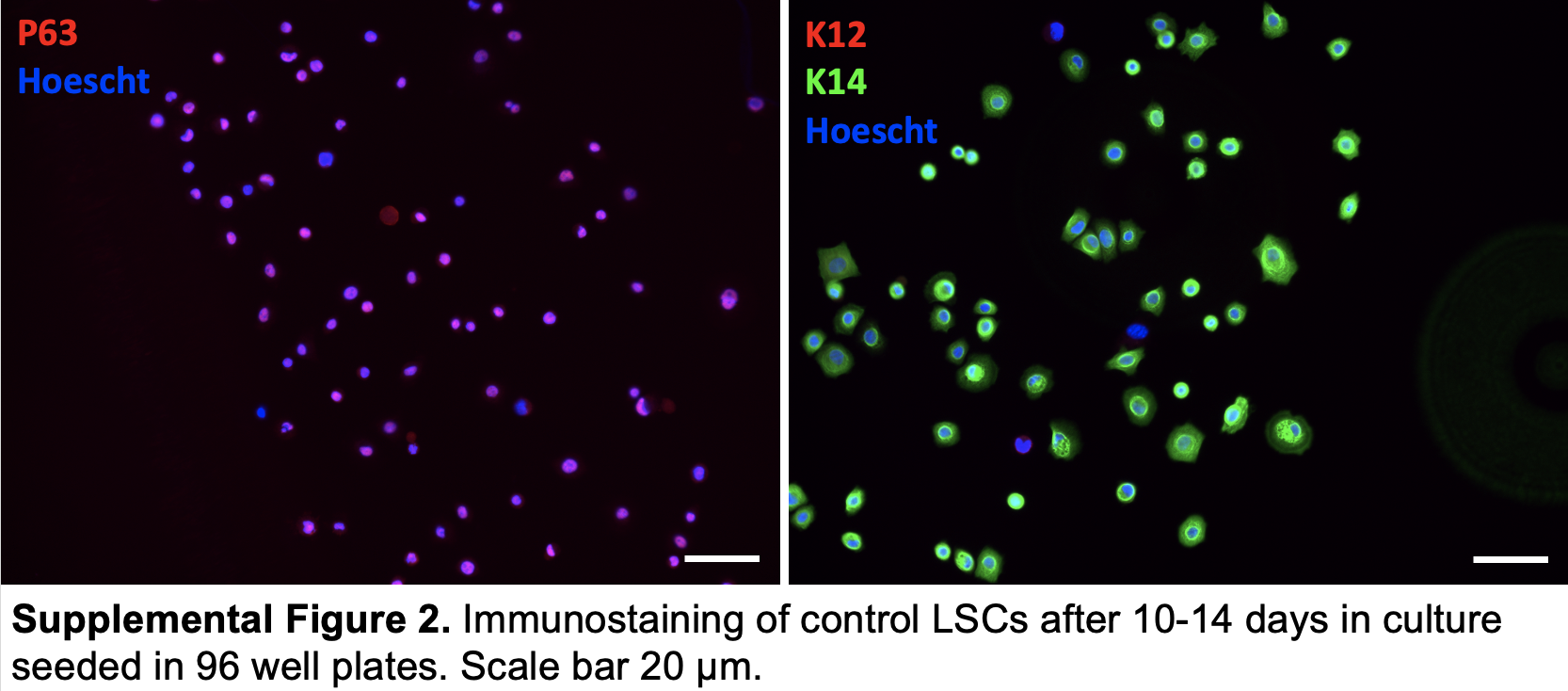

Supplement: Supplementary file 2 — Supplementary Figure 2. [file 41598_2023_42794_MOESM2_ESM.tiff]
