# Supplementary material for: Wnt activation as a potential therapeutic approach to treat partial limbal stem cell deficiency
Source: Sci Rep. 2023 Sep 21;13:15670. doi: 10.1038/s41598-023-42794-8 (PMC10514048; doi:10.1038/s41598-023-42794-8)
Supplement: Supplementary file 3 — Supplementary Table 1. [file 41598_2023_42794_MOESM3_ESM.docx]

Supplemental Table 1. Primary and secondary antibodies

| Antibody | Brand | Host | Target | Dilution |
| --- | --- | --- | --- | --- |
| Anti-P63α | Cell Signaling #4892 | Rabbit | Human | 1:100 |
| Anti-K12 | Santa Cruz Bio #sc-25722 | Mouse | Human | 1:100 |
| Anti-K14 | ThermoFisher #MS-115-R7 | Rabbit | Human | 1:2 |
| Vimentin | Sigma-Aldrich #V6389 | Mouse | Human | 1:200 |
| Pan-cytokeratin | Dako-Omnis #GA05361 | Mouse | Human | 1:200 |
| Alexa Fluor 546 IgG | Invitrogen #A11035 | Goat | Rabbit | 1:500 |
| Alexa Fluor 488 IgG | Invitrogen #A11029 | Goat | Mouse | 1:500 |
| Alexa Fluor 488 IgG | Invitrogen # A48269 | Donkey | Rat | 1:500 |
